# Integrating Topological Proofs with Model Checking to Instrument Iterative Design

**DOI:** 10.1007/978-3-030-45234-6_3

**Published:** 2020-03-13

**Authors:** Claudio Menghi, Alessandro Maria Rizzi, Anna Bernasconi

**Affiliations:** 8 University of Paderborn, Paderborn, Germany; 9 ICREA, Open University of Catalonia, Barcelona, Spain; 10 University of Luxembourg, Centre for Security Reliability and Trust (SnT), 29 Avenue John F.Kennedy, 1855 Luxembourg, Luxembourg; 11 Politecnico di Milano, Milano, Italy

**Keywords:** Topological Proofs, Iterative Design, Model Checking, Theorem Proving, Unsatisfiable Core

## Abstract

System development is not a linear, one-shot process. It proceeds through refinements and revisions. To support assurance that the system satisfies its requirements, it is desirable that continuous verification can be performed after each refinement or revision step. To achieve practical adoption, formal verification must accommodate continuous verification efficiently and effectively. Model checking provides developers with information useful to improve their models only when a property is not satisfied, i.e., when a counterexample is returned. However, it is desirable to have some useful information also when a property is instead satisfied. To address this problem we propose TOrPEDO, an approach that supports verification in two complementary forms: model checking and proofs. While model checking is typically used to pinpoint model behaviors that violate requirements, proofs can instead explain why requirements are satisfied. In our work, we introduce a specific notion of proof, called Topological Proof. A topological proof produces a slice of the original model that justifies the property satisfaction. Because models can be incomplete, TOrPEDO supports reasoning on requirements satisfaction, violation, and possible satisfaction (in the case where satisfaction depends on unknown parts of the model). Evaluation is performed by checking how topological proofs support software development on 12 modeling scenarios and 15 different properties obtained from 3 examples from literature. Results show that: (i) topological proofs are $\approx$60% smaller than the original models; (ii) after a revision, in $\approx$78% of cases, the property can be re-verified by relying on a simple syntactic check.

## 1 Introduction

One of the goals of software engineering and formal methods is to provide automated verification tools that support designers in producing models of an envisioned system which follows a set of properties of interest. Designers benefit from automated support to understand why their system does not behave as expected (e.g., counterexamples), but they might find it also useful to retrieve information when the system already follows the specified requirements. While model checkers provide the former, theorem provers sustain the latter. Theorem provers usually rely on some form of deductive mechanism that, given a set of axioms, iteratively applies a set of rules until a theorem is proved. The proof consists of the specific sequence of deductive rules applied to prove the theorem. In the literature, many approaches have dealt with an integration of model checking and theorem proving at various levels (e.g., [48,60,53,36]). These approaches are oriented to produce *certified model checking* procedures rather than tools that actually help the design process. Even when the idea is to provide a practically useful framework [49,50], the output consists of deductive proofs that are usually difficult to understand and hardly connectable with the designer's modeling choices. Moreover, verification techniques only take into account completely specified designs. This is a remarkable limitation in practical contexts, where the designer may start by providing an initial, high-level version of the model, which is iteratively narrowed down as design progresses and uncertainties are removed [13,42,8,19,65,43]. A recent work [4,5] considered cases in which a partial knowledge of the system model is available. However, the presented approach was mainly theoretical and lacked a practical implementation.

We formulate our problem on models that contain uncertain parts. We choose Partial Kripke Structures (PKSs) as a formalism to represent general models for the following reasons: (i) PKSs have been used in requirement elicitation to reason about system behavior from different points of view [19,8], and are a common theoretical reference language used in the formal method community for the specification of uncertain models (e.g, [26,9,27,10]); (ii) other modeling formalisms commonly used in software development [23,64], such as Modal Transition Systems [37] (MTSs), can be converted into PKSs through a simple transformation [26] making our solution easily applicable to those models.

Kripke Structures (KSs) are particular instances of PKSs that represent complete models. Requirements on the model are expressed in Linear-time Temporal Logic (LTL). As such, the approach presented in the following is generic: it can be applied on models that contain uncertain parts (PKSs) or not (KSs), and can be easily adapted to support MTSs.

Verification techniques that consider PKSs return three alternative values: *true* if the property holds in the partial model, *false* if it does not hold, and *maybe* if the property possibly holds, i.e., its satisfaction depends on the parts of the model that still need to be refined. As models are revised, i.e., they are modified during design iterations, designers need support to understand *why* properties are satisfied, or possibly satisfied.

A comprehensive and integrated design framework able to support software designers in understanding such motivation is still missing. We tackle this problem by presenting TOrPEDO (TOpological Proof drivEn Development frameWOrk), a novel automated verification framework, that:

(i) supports a modeling formalism which allows a partial specification of the system design;

(ii) allows performing analysis and verification in the context of systems in which "incompleteness" represents a conceptual uncertainty;
(iii) provides guidance in producing model revisions through complementary outputs: counterexamples and proofs;
(iv) when the system is completely specified, allows understanding which changes impact or not the satisfaction of certain properties.

TOrPEDO is based on the novel notion of *topological proof* (TP), which tries to overcome the complexity of deductive proofs and is designed to make proofs understandable on the original system design. A TP is a *slice* of the original model that specifies which part of it impacts the property satisfaction. If the slice defined by the TP is not preserved during a revision, there is no assurance that the property holds (possibly holds) in the revised model. This paper proposes an algorithm to compute topological proofs—which relies on the notion of *unsatisfiable cores* (UCs) [56]—and proves its correctness on PKSs. It also proposes an algorithm that checks whether a TP is preserved in a model revision. This simple syntactic check avoids (in many cases) the execution of the model checking procedure. While architectural decomposition and composition of components can be considered during the system development [42], in this work we present our solution by assuming that the system is modeled as a single PKS. However, our framework can be extended to consider the composition of components, such as the parallel composition of PKSs or MTSs. This can be done by extracting the portions of the TP that refer to the different components.

TOrPEDO has been implemented on top of NuSMV [14] and PLTL-MUP [58]. The implementation has been exploited to evaluate TOrPEDO by considering a set of examples coming from literature including both completely specified and partially specified models. We considered 3 different example models and 4 variations for each model that was presented in the literature [12,20]. We considered 15 properties, i.e., 5 for each example, leading to a total of 60 (3×4×5) scenarios that require the evaluation of a property on a model. We evaluated how our framework supports model design by comparing the size of the generated topological proofs against the size of the original models. Results show that topological proofs are ≈60% smaller than the original models. Moreover, after a revision, in ≈78% of cases, our syntactic check avoids the re-execution of the model checker.

**Organization.** Section 2 describes TOrPEDO. Section 3 discusses the background. Sections 4 and 5 present the theoretical results and the algorithms that support TOrPEDO. Section 6 evaluates the achieved results. Section 7 discusses related work. Section 8 concludes.

## 2 TOrPEDO

TOrPEDO is a proof based development framework which allows verifying PKSs and evaluating their revisions. To illustrate TOrPEDO, we use a simple model describing the states of a vacuum-cleaner robot that has to satisfy the requirements in Fig. 2, specified through LTL formulae and English natural language.

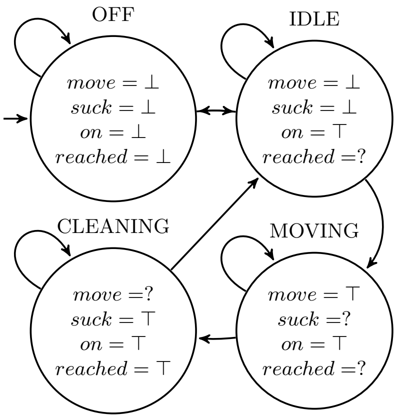

Fig. 1: PKS of a vacuum-cleaner robot.

| **LTL formulae** |
|---|
| $\phi_1 = \mathcal{G}(suck \rightarrow reached)$ |
| $\phi_2 = \mathcal{G}((\neg move)\,\mathcal{W}\,on)$ |
| $\phi_3 = \mathcal{G}(((\neg move) \wedge on) \rightarrow suck)$ |
| $\phi_4 = ((\neg suck)\,\mathcal{W}(move \wedge (\neg suck)))$ |
| **Textual requirements** |
| $\phi_1$: the robot is drawing dust (*suck*) only if it has *reached* the cleaning site. |
| $\phi_2$: the robot must be turned *on* before it can *move*. |
| $\phi_3$: if the robot is *on* and stationary ($\neg move$), it must be drawing dust (*suck*). |
| $\phi_4$: the robot must *move* before it is allowed to draw dust (*suck*). |

Fig. 2: Natural language and LTL formulation of the requirements of the vacuum-cleaner robot. $\mathcal{G}$ and $\mathcal{W}$ are the "globally" and "weak until" LTL operators.

The TOrPEDO framework is illustrated in Fig. 3 and carries out verification in four phases: INITIAL DESIGN, ANALYSIS, REVISION, and RE-CHECK.

**INITIAL DESIGN (1).** The model of the system is expressed using a PKS $M$ (1), which can be generated from other languages, along with the property of interest $\phi$, in LTL (2).

*Running example.* The PKS presented in Fig. 1 is defined over two atomic propositions representing actions that a robot can perform: *move*, i.e., the agent travels to the cleaning site; *suck*, i.e., the agent is drawing the dust, and two atomic propositions representing conditions that can trigger actions: *on*, true when the robot is turned on; *reached*, true when the robot has reached the cleaning site. The state *OFF* represents the robot being shut down, *IDLE* the robot being tuned on w.r.t. a cleaning call, *MOVING* the robot reaching the cleaning site, and *CLEANING* the robot performing its duty. Each state is labeled with the actions *move* and *suck* and the conditions *on* and *reached*. Given an action or condition $\alpha$ and a state $s$, we use the notation: $\alpha = \top$ to indicate that $\alpha$ occurs when the robot is in state $s$; $\alpha = \bot$ to indicate that $\alpha$ does not occur when the robot is in state $s$; $\alpha =?$ to indicate that there is uncertainty on whether $\alpha$ occurs when the robot is in state $s$.

**ANALYSIS (2).** TOrPEDO provides automated analysis support, which includes the following elements:

(i) Information about *what is wrong* in the current design. This information includes a definitive-counterexample, which indicates a behavior that depends on already performed design choices and violates the properties of interest. The definitive-counterexample (3 ⊥-CE) can be used to produce a revised version $M'$ of $M$ that satisfies or possibly satisfies the property of interest.

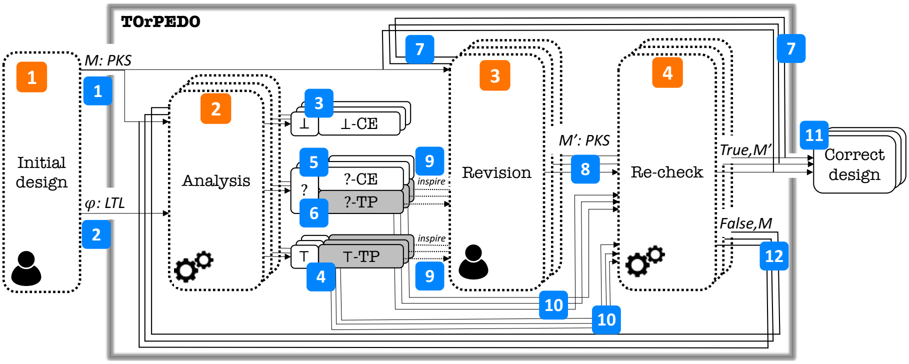

Fig. 3: TOrPEDO structure. Continuous arrows represent inputs and outputs to phases. Numbers are used to reference the image in the text.

(ii) Information about *what is correct* in the current design. This information includes definitive-topological proofs (4 ⊤-TP) that indicate a portion of the design that ensures satisfaction of the property.

(iii) Information about *what could be wrong/correct* in the current design, depending on how uncertainty is removed. This information includes: a possible-counterexample (5 ?-CE), indicating a behavior (which depends on uncertain actions and conditions) that violates the properties of interest, and a possible-topological proof (6 ?-TP), indicating a portion of the design that ensures the possible satisfaction of the property of interest.

In the following we will use the notation $x$-topological proofs or $x$-TP to indicate arbitrarily definitive-topological or possible-topological proofs. The results returned by TORPEDO for the different properties in our motivating example are presented in Table 1. Property $\phi_2$ is satisfied, $\phi_3$ is not. In those cases TORPEDO returns respectively a definitive-proof and a definitive-counterexample. Since $\phi_1$ and $\phi_4$ are possibly satisfied, in both cases a possible-counterexample and a possible-topological proof are returned.

*Running example.* For $\phi_1$ the possible-counterexample shows a run that may violate the property of interest. The possible-topological proof in Table 1 shows that if *OFF* remains the only initial state (**TPI**), *reached* still holds in *CLEANING*, and *suck* does not hold in *OFF* and *IDLE*, while unknown in *MOVING* (**TPP**), property $\phi_1$ remains possibly satisfied. In addition, all transitions must be preserved (**TPT**).[3] Note that the proof highlights portions of the model that influence the property satisfaction. For example, by inspecting the proof, the designer understands that she can change the value of the proposition *reached* in all the states of the PKS, with the exception of the state *CLEANING*, without making the property violated.

[3] The precise formal descriptions of $x$-topological proofs, **TPI**, **TPT** and **TPT** are presented in Section 4.

Table 1: Results provided by TOrPEDO for properties $\phi_1$, $\phi_2$, $\phi_3$ and $\phi_4$. $\top$, $\bot$ and ? indicate that the property is satisfied, violated and possibly satisfied.

| | | | |
|---|---|---|---|
| $\phi_1$ | ? | **?-CE** | $\mathit{OFF}$, $\mathit{IDLE}$, $(\mathit{MOVING})^\omega$. |
| | | **?-TP** | **TPP**: $\langle \mathit{CLEANING}, \mathit{reached}, \top\rangle$ $\langle \mathit{OFF}, \mathit{suck}, \bot\rangle$, $\langle \mathit{IDLE}, \mathit{suck}, \bot\rangle$, $\langle \mathit{MOVING}, \mathit{suck}, ?\ \rangle$<br>**TPT**: $\langle \mathit{OFF}, \{\mathit{OFF}, \mathit{IDLE}\}\rangle$, $\langle \mathit{IDLE}, \{\mathit{OFF}, \mathit{IDLE}, \mathit{MOVING}\}\rangle$, $\langle \mathit{MOVING}, \{\mathit{MOVING}, \mathit{CLEANING}\}\rangle$, $\langle \mathit{CLEANING}, \{\mathit{CLEANING}, \mathit{IDLE}\}\rangle$<br>**TPI**: $\langle\{\mathit{OFF}\}\rangle$ |
| $\phi_2$ | $\top$ | **$\top$-TP** | **TPP**: $\langle \mathit{MOVING}, \mathit{on}, \top\rangle$, $\langle \mathit{CLEANING}, \mathit{on}, \top\rangle$, $\langle \mathit{OFF}, \mathit{move}, \bot\ \rangle$, $\langle \mathit{IDLE}, \mathit{move}, \bot\rangle$<br>**TPT**: $\langle \mathit{OFF}, \{\mathit{OFF}, \mathit{IDLE}\}\rangle$, $\langle \mathit{IDLE}, \{\mathit{OFF}, \mathit{IDLE}, \mathit{MOVING}\}\rangle$, $\langle \mathit{MOVING}, \{\mathit{MOVING}, \mathit{CLEANING}\}\rangle$, $\langle \mathit{CLEANING}, \{\mathit{CLEANING}, \mathit{IDLE}\}\rangle$<br>**TPI**: $\langle\{\mathit{OFF}\}\rangle$ |
| $\phi_3$ | $\bot$ | **$\bot$-CE** | $\mathit{OFF}, \mathit{IDLE}^\omega$ |
| $\phi_4$ | ? | **?-CE** | $\mathit{OFF}$, $(\mathit{IDLE}$, $\mathit{MOVING}$, $\mathit{CLEANING}$, $\mathit{IDLE}$, $\mathit{OFF})^\omega$ |
| | | **?-TP** | **TPP**: $\langle \mathit{OFF}, \mathit{suck}, \bot\rangle$, $\langle \mathit{IDLE}, \mathit{suck}, \bot\rangle$, $\langle \mathit{MOVING}, \mathit{suck}, ?\ \rangle$, $\langle \mathit{MOVING}, \mathit{move}, \top\rangle$<br>**TPT**: $\langle \mathit{OFF}, \{\mathit{OFF}, \mathit{IDLE}\}\rangle$, $\langle \mathit{IDLE}, \{\mathit{OFF}, \mathit{IDLE}, \mathit{MOVING}\}\rangle$<br>**TPI**: $\langle\{\mathit{OFF}\}\rangle$ |

**Revision (3).** Revisions (8) can be obtained by changing some parts of the model: adding/removing states and transitions or by changing propositions labelling inside states, and are defined by considering the TP (9).

*Running example.* The designer may want to propose a revision that still does not violate properties $\phi_1$, $\phi_2$, and $\phi_4$. Thus, she changes the values of some atomic propositions: *move* becomes $\top$ in state *CLEANING* and *reached* becomes $\bot$ in state *IDLE*. Since $\phi_1$, $\phi_2$, and $\phi_4$ were previously not violated, TOrPEDO performs the RE-CHECK phase for each property.

**Re-check (4).** The automated verification tool provided by TOrPEDO checks whether all the changes in the current model revision are compliant with the $x$-TPs (10), i.e., changes applied to the revised model do not include parts that had to be preserved according to the $x$-topological proof. If a property of interest is (possibly) satisfied in a previous model, and the revision of the model is compliant with the property $x$-TP, the designer has the guarantee that the property is (possibly) satisfied in the revision. Thus, she can perform another model revision round (7) or approve the current design (11). Otherwise, TOrPEDO re-executes the ANALYSIS (12).

*Running example.* In the vacuum-cleaner case, the revision passes the RE-CHECK and the designer proceeds to a new revision phase.

## 3 Background

We present background notions by relying on standard notations for the selected formalisms (see for example [26,9,10,30]).

**Partial Kripke Structures (1)** are state machines that can be adopted when the value of some propositions is uncertain on selected states.

**Definition 1 ([9],[35]).** *A* Partial Kripke Structure *(PKS) $M$ is a tuple $\langle S, R, S_0, AP, L\rangle$, where: $S$ is a set of states; $R \subseteq S \times S$ is a left-total transition*

*relation on $S$; $S_0$ is a set of initial states; $AP$ is a set of atomic propositions; $L : S \times AP \to \{\top, ?, \bot\}$ is a function that, for each state in $S$, associates a truth value to every atomic proposition in $AP$. A* Kripke Structure *(KS) $M$ is a PKS $\langle S, R, S_0, AP, L\rangle$, where $L : S \times AP \to \{\top, \bot\}$.*

A PKS represents a system as a set of states and transitions between these states. Uncertainty on the $AP$ is represented through the value ?. The model in Fig. 1 is a PKS where propositions in $AP$ are used to model actions and conditions.

**LTL properties (2).** For KSs we consider the classical LTL semantics $[M \models \phi]$ over infinite words that associates to a model $M$ and a formula $\phi$ a truth value in the set $\{\bot, \top\}$. The interested reader may refer, for example, to [3]. Let $M$ be a KS and $\phi$ be an LTL property. We assume that the function CHECK, such that $\langle res, c\rangle = \text{CHECK}(M, \phi)$, returns a tuple $\langle res, c\rangle$, where $res$ is the model checking result in $\{\top, \bot\}$ and $c$ is the counterexample if $res = \bot$, else an empty set.

The *three-valued LTL semantics* [9] $[M \models \phi]$ associates to a model $M$ and a formula $\phi$ a truth value in the set $\{\bot, ?, \top\}$ and is defined based on the information ordering $\top > ? > \bot$. The three-valued LTL semantics is defined by considering paths of the model $M$. A path $\pi$ is an infinite sequence of states $s_0, s_1, \ldots$ such that, for all $i \geq 0$, $(s_i, s_{i+1}) \in R$. We use the symbol $\pi^i$ to indicate the infinite sub-sequence of $\pi$ that starts at position $i$, and $Path(s)$ to indicate all the paths that start in the state $s$.

**Definition 2 ([9]).** *Let $M = \langle S, R, S_0, AP, L\rangle$ be a PKS, $\pi = s_0, s_1, \ldots$ be a path, and $\phi$ be an LTL formula. Then, the* three-valued semantics $[(M, \pi) \models \phi]$ *is defined inductively as follows:*

$$\begin{array}{lcl}
[(M,\pi) \models p] & = & L(s_0, p) \\
[(M,\pi) \models \neg\phi] & = & comp([(M,\pi) \models \phi]) \\
[(M,\pi) \models \phi_1 \wedge \phi_2] & = & \min([(M,\pi) \models \phi_1], [(M,\pi) \models \phi_2]) \\
[(M,\pi) \models \mathcal{X}\,\phi] & = & [(M,\pi^1) \models \phi] \\
[(M,\pi) \models \phi_1 \,\mathcal{U}\, \phi_2] & = & \max\limits_{j \geq 0}(\min(\{[(M,\pi^i) \models \phi_1] | i < j\} \cup \{[(M,\pi^j) \models \phi_2]\}))
\end{array}$$

*Let $M = \langle S, R, S_0, AP, L\rangle$ be a PKS, and $\phi$ be an LTL formula. Then $[M \models \phi] = \min(\{[(M,\pi) \models \phi] \mid \pi \in Path(s)$ and $s \in S_0\})$.*

The conjunction (resp. disjunction) is defined as the minimum (resp. maximum) of its arguments, following the order $\bot < ? < \top$. These functions are extended to sets with $\min(\emptyset)=\top$ and $\max(\emptyset)=\bot$. The *comp* operator maps $\top$ to $\bot$, $\bot$ to $\top$, and ? to ?. The semantics of the $\mathcal{G}$ ("globally") and $\mathcal{W}$ ("weak until") operators is defined as usual [28].

**Model Checking.** Checking KSs with respect to LTL properties can be done by using classical model checking procedures. For example, the model checking problem of property $\phi$ on a KS $M$ can be reduced to the satisfiability problem of the LTL formula $\Phi_M \wedge \neg\phi$, where $\Phi_M$ represents the behaviors of model $M$. If $\Phi_M \wedge \neg\phi$ is satisfiable, then $[M \models \phi] = \bot$, otherwise $[M \models \phi] = \top$.

Checking a PKS $M$ with respect to an LTL property $\phi$ considering the three-valued semantics is done by performing twice the classical model checking procedure for KSs [10], one considering an optimistic approximation $M_{opt}$ and one considering a pessimistic approximation $M_{pes}$. These two procedures consider the LTL formula $\phi' = \mathtt{F}(\phi)$, where $\mathtt{F}$ transforms $\phi$ with the following steps: (i) negate $\phi$; (ii) convert $\neg\phi$ in negation normal form; (iii) replace every subformula $\neg\alpha$, where $\alpha$ is an atomic proposition, with a new proposition $\overline{\alpha}$.

To create the optimistic and pessimistic approximations $M_{opt}$ and $M_{pes}$, the PKS $M = \langle S, R, S_0, AP, L\rangle$ is first converted into its *complement-closed* version $M_c = \langle S, R, S_0, AP_c, L_c\rangle$ where the set of atomic propositions $AP_c = AP \cup \overline{AP}$ is such that $\overline{AP} = \{\overline{\alpha} \mid \alpha \in AP\}$. Atomic propositions in $\overline{AP}$ are called complement-closed propositions. Function $L_c$ is such that, for all $s \in S$ and $\alpha \in AP$, $L_c(s, \alpha) = L(s, \alpha)$ and, for all $s \in S$ and $\overline{\alpha} \in \overline{AP}$, $L_c(s, \overline{p}) = comp(L(s, p))$. The complement-closed PKS of the vacuum-cleaner agent in Fig. 1 presents eight propositional assignments in the state $IDLE$: $move = \bot$, $\overline{move} = \top$, $suck = \bot$, $\overline{suck} = \top$, $on = \top$, $\overline{on} = \bot$, $reached =?$, and $\overline{reached} =?$.

The two model checking runs for a PKS $M = \langle S, R, S_0, AP, L\rangle$ are based respectively on an optimistic ($M_{opt} = \langle S, R, S_0, AP_c, L_{opt}\rangle$) and a pessimistic ($M_{pes} = \langle S, R, S_0, AP_c, L_{pes}\rangle$) approximation of $M$'s related complement-closed $M_c = \langle S, R, S_0, AP_c, L_c\rangle$. Function $L_{pes}$ (resp. $L_{opt}$) is such that, for all $s \in S$, $\alpha \in AP_c$, and $L_c(s, \alpha) \in \{\top, \bot\}$, then $L_{pes}(s, \alpha) = L_c(s, \alpha)$ (resp. $L_{opt}(s, \alpha) = L_c(s, \alpha)$), and, for all $s \in S$, $\alpha \in AP_c$, and $L_c(s, \alpha) =?$, then $L_{pes}(s, \alpha) = \bot$ (resp. $L_{opt}(s, \alpha) = \top$).

Let $\mathcal{A}$ be a KS and $\phi$ be an LTL formula, $\mathcal{A} \models^* \phi$ is true if no path that satisfies the formula $\mathtt{F}(\phi)$ is present in $\mathcal{A}$.

**Theorem 1 ([9]).** *Let $\phi$ be an LTL formula, let $M = \langle S, R, S_0, AP, L\rangle$ be a PKS, and let $M_{pes}$ and $M_{opt}$ be the pessimistic and optimistic approximations of $M$'s relative complement-closed $M_c$. Then*

$$[M \models \phi] \stackrel{def}{=} \begin{cases} \top & \textit{if } M_{pes} \models^* \phi \\ \bot & \textit{if } M_{opt} \not\models^* \phi \\ ? & \textit{otherwise} \end{cases} \tag{1}$$

We call Check* the function that computes the result of operator $\models^*$. It takes as input either $M_{pes}$ or $M_{opt}$ and the property $\mathtt{F}(\phi)$, and returns a tuple $\langle res, c\rangle$, where $res$ is the model checking result in $\{\top, \bot\}$, and $c$ can be an empty set (when $M$ satisfies $\phi$), a *definitive*-counterexample (3, when $M$ violates $\phi$), or a *possible*-counterexample (5, when $M$ possibly-satisfies $\phi$).

## 4 Revising models

We define how models can be revised and the notion of *topological proof*, that is used to describe why a property $\phi$ is (possibly) satisfied in a PKS $M$.

**Initial design and revisions (1,3).** In the initial design a preliminary PKS is manually defined or automatically obtained from other modeling formalisms.

During a revision, a designer can add and remove states and transitions and/or change the labeling of the atomic propositions in the states of the PKS. Let $M = \langle S, R, S_0, AP, L\rangle$ and $M' = \langle S', R', S'_0, AP', L'\rangle$ be two PKSs. Then $M'$ is a *revision* of $M$ if and only if $AP \subseteq AP'$. Informally, the only constraint the designer has to respect during a revision is not to remove propositions from the set of atomic propositions. This condition is necessary to ensure that any property that can be evaluated on $M$ can also be evaluated on $M'$, i.e., every atomic proposition has a value in each of the states of the automaton. The deactivation of a proposition can instead be simulated by associating its value to $\bot$ in all the states of $M'$.

**Topological proofs ([4,6]).** The pursued proof is made of a set of clauses specifying certain topological properties of $M$, which ensure that the property is (possibly) satisfied.

**Definition 3.** *Let $M = \langle S, R, S_0, AP, L\rangle$ be a PKS. A* Topological Proof clause *(TP-clause) $\gamma$ for $M$ is either:*

- *a* Topological Proof Propositional clause *(TPP-clause), i.e., a triad $\langle s, \alpha, v\rangle$ where $s \in S$, $\alpha \in AP$, and $v \in \{\top, ?, \bot\}$;*
- *a* Topological Proof Transitions-from-state clause *(TPT-clause), i.e., a pair $\langle s, T\rangle$, such that $s \in S, T \subseteq S$;*
- *a* Topological Proof Initial-states clause *(TPI-clause), i.e., an element $\langle S_0\rangle$.*

These clauses indicate *topological properties* of a PKS $M$. Informally, TPP-clauses constrain how states are labeled ($L$), TPT-clauses constrain how states are connected ($R$), and TPI-clauses constrain from which states the runs on the model begin ($S_0$). For example, in Table 1, for property $\phi_1$, $\langle$*CLEANING*, *reached*, $\top\rangle$ is a TPP-clause that constrains the atomic proposition *reached* to be labeled as true ($\top$) in the state *CLEANING*; $\langle$*OFF*, $\{$*OFF*, *IDLE*$\}\rangle$ is a TPT-clause that constrains the transition from *OFF* to *OFF* and from *OFF* to *IDLE* to not be removed; and $\langle\{$*OFF*$\}\rangle$ is a TPI-clause that constrains the state *OFF* to remain the initial state of the system.

A state $s'$ is constrained: by a TPP-clause $\langle s, \alpha, v\rangle$ if $s = s'$, by a TPT-clause $\langle s, T\rangle$ if $s = s'$ or $s' \in T$, and by a TPI-clause $\langle S_0\rangle$ if $s' \in S_0$.

**Definition 4.** *Let $M = \langle S, R, S_0, AP, L\rangle$ be a PKS and let $\Omega$ be a set of TP-clauses for $M$. Then a PKS $\Omega$-*related *to $M$ is a PKS $M' = \langle S', R', S'_0, AP', L'\rangle$, such that the following conditions hold:*

- *$AP \subseteq AP'$;*
- *for every TPP-clause $\langle s, \alpha, v\rangle \in \Omega$, $s \in S'$, $v = L'(s, \alpha)$;*
- *for every TPT-clause $\langle s, T\rangle \in \Omega$, $s \in S'$, $T \subseteq S'$, $T = \{s' \in S' | (s, s') \in R'\}$;*
- *for every TPI-clause $\langle S_0\rangle \in \Omega$, $S_0 = S'_0$.*

Intuitively, a PKS $\Omega$-related to $M$ is a PKS obtained from $M$ by changing any topological aspect that does not impact on the set of TP-clauses $\Omega$. Any transition whose source state is not the source state of a transition included in the TPT-clauses can be added or removed from the PKS and any value of a proposition that is not constrained by a TPP-clause can be changed. States can be always added and they can be removed if they are not constrained by any TPT-, TPP-, or TPI-clause. Initial states cannot be changed if $\Omega$ contains a TPI-clause.

**Definition 5.** *Let $M = \langle S, R, S_0, AP, L \rangle$ be a PKS, let $\phi$ be an LTL property, let $\Omega$ be a set of TP-clauses, and let $x$ be a truth value in $\{\top, ?\}$. A set of TP-clauses $\Omega$ is an $x$-*topological proof *(or $x$-TP) for $\phi$ in $M$ if: (i) $[M \models \phi] = x$; and (ii) every PKS $M'$ $\Omega$-related to $M$ is such that $[M' \models \phi] \geq x$.*

Intuitively, an *$x$-topological proof* is a set of TP-clauses $\Omega$ such that every PKS $M'$ that satisfies the conditions specified in Definition 4 is such that $[M' \models \phi] \geq x$. We call $\top$-TP a *definitive-topological proof* and ?-TP a *possible-topological proof*. In Definition 5, the operator $\geq$ assumes that values $\top, ?, \bot$ are ordered considering the classical information ordering $\top > ? > \bot$ among the truth values [9].

Regarding the PKS in Fig. 1, Table 1 shows two ?-TPs for properties $\phi_1$ and $\phi_4$, and one $\top$-TP for property $\phi_2$.

**Definition 6.** *Let $M$ and $M'$ be two PKSs, let $\phi$ be an LTL property, and let $\Omega$ be an $x$-TP. Then $M'$ is an $\Omega_x$-*revision *of $M$ if $M'$ is $\Omega$-related to $M$.*

Intuitively, since the *$\Omega_x$-revision* $M'$ of $M$ is such that $M'$ is $\Omega$-related to $M$, it is obtained by changing the model $M$ while preserving the statements that are specified in the $x$-TP. A revision $M'$ of $M$ is *compliant* with the $x$-TP for a property $\phi$ in $M$ if it is an *$\Omega_x$-revision* of $M$.

**Theorem 2.** *Let $M$ be a PKS, let $\phi$ be an LTL property such that $[M \models \phi] = \top$, and let $\Omega$ be a $\top$-TP. Then every $\Omega_\top$-revision $M'$ is such that $[M' \models \phi] = \top$. Let $M$ be a PKS, let $\phi$ be an LTL property such that $[M \models \phi] = ?$, and let $\Omega$ be an ?-TP. Then every $\Omega_?$-revision $M'$ is such that $[M' \models \phi] \in \{\top, ?\}$.*

*Proof Sketch.* We prove the first statement of the Theorem; the proof of the second statement is obtained by following the same steps.

If $\Omega$ is a $\top$-TP, it is a $\top$-TP for $\phi$ in $M'$, since $M'$ is an $\Omega_\top$-revision of $M$ (by Definition 6). Furthermore, since $\Omega$ is a $\top$-TP for $\phi$ in $M'$, then $[M' \models \phi] \geq \top$ (by Definition 5). □

## 5 TOrPEDO automated support

This section describes the algorithms that support the ANALYSIS and RE-CHECK phases of TOrPEDO.

**ANALYSIS (2).** To analyze a PKS $M = \langle S, R, S_0, AP, L \rangle$ (1), TOrPEDO uses the three-valued model checking framework based on Theorem 1. The model checking result is provided as output by the ANALYSIS phase of TOrPEDO, whose behavior is described in Algorithm 1.

1: **function** ANALYZE($M$, $\phi$)
2: $\langle res, c\rangle$ = CHECK*($M_{opt}$, $\phi$)
3: **if** $res == \bot$ **then return** $\langle \bot, \{c\}\rangle$
4: **else**
5: $\langle res', c'\rangle$ = CHECK*($M_{pes}$, $\phi$)
6: **if** $res' == \top$ **then return**
7: $\langle \top, \{$CTP_KS($M, M_{pes}$, F($\phi$))$\}\rangle$
8: **else**
9: **return**
10: $\langle ?, \{c'$, CTP_KS($M, M_{opt}$, F($\phi$))$\}\rangle$
11: **end if**
12: **end if**
13: **end function**

Algorithm 1: The ANALYSIS algorithm.

1: **function** CTP_KS($M$, $\mathcal{A}$, $\psi$)
2: $\eta(C_{\mathcal{A}} \cup \{\psi\})$ = SYS2LTL($\mathcal{A}, \psi$)
3: $\eta(C'_{\mathcal{A}} \cup \{\psi\})$ = GETUC($\eta(C_{\mathcal{A}} \cup \{\psi\})$)
4: $TP$ = GETTP($M, \eta(C'_{\mathcal{A}} \cup \{\psi\})$)
5: **return** $TP$
6: **end function**

Algorithm 2: Compute Topological Proofs.

The algorithm returns a tuple $\langle x, y\rangle$, where $x$ is the verification result and $y$ is a set containing the counterexample, the topological proof or both of them. The algorithm first checks whether the optimistic approximation $M_{opt}$ of the PKS $M$ satisfies property $\phi$ (2, Line 2). If this is not the case, the property is violated by the PKS and the definitive-counterexample $c$ (3, $\bot$-CE) is returned (Line 3). Then, it checks whether the pessimistic approximation $M_{pes}$ of the PKS $M$ satisfies property $\phi$ (Line 5). If this is the case, the property is satisfied and the value $\top$ is returned along with the definitive-topological proof (4, $\top$-TP) computed by the CTP_KS procedure applied on the pessimistic approximation $M_{pes}$ and the property F($\phi$) (Line 7).

If this is not the case, the property is possibly satisfied and the value ? is returned along with the possible-counterexample $c'$ (5, ?-CE) and the possible-topological proof (6, ?-TP) computed by the CTP_KS procedure applied to $M_{opt}$ and F($\phi$) (Line 10).

The procedure CTP_KS (Compute Topological Proofs) to compute $x$-TPs is described in Algorithm 2. It takes as input a PKS $M$, its optimistic/pessimistic approximation, i.e., denoted generically as the KS $\mathcal{A}$, and an LTL formula $\psi$— satisfied in $\mathcal{A}$— corresponding to the transformed property F($\phi$) (see Section 3). The three steps of the algorithm are described in the following.

SYS2LTL. *Encoding of the KS $\mathcal{A}$ and the LTL formula $\psi$ into an LTL formula $\eta(C_{\mathcal{A}} \cup \{\psi\})$.* The KS $\mathcal{A} = \langle S, R, S_0, AP_c, L_{\mathcal{A}}\rangle$ (where $L_{\mathcal{A}}$ is the optimistic or pessimistic function, $L_{opt}$ or $L_{pes}$, as defined in Section 3) and the LTL formula $\psi$ are used to generate an LTL formula

$$\eta(C_{\mathcal{A}} \cup \{\psi\}) = \bigwedge_{c \in (C_{\mathcal{A}} \cup \{\psi\})} c$$

Table 2: Rules to transform the KS in LTL formulae.

| |
|---|
| $c_i = \bigvee_{s \in S_0} p(s)$<br>The KS is initially in one of its initial states. |
| $CR = \{\mathcal{G}(\neg p(s) \vee \mathcal{X}(\bigvee_{(s,s') \in R} p(s'))) \mid s \in S\}$<br>If the KS is in state $s$ in the current instant, in the next instant it is in one of the successors $s'$ of $s$. |
| $CL_{\top,\mathcal{A}} = \{\mathcal{G}(\neg p(s) \vee \alpha) \mid s \in S, \alpha \in AP_c, L_{\mathcal{A}}(s,\alpha) = \top\}$<br>If the KS is in state $s$ s.t. $L_{\mathcal{A}}(s,\alpha) = \top$, the atomic proposition $\alpha$ is true. |
| $CL_{\bot,\mathcal{A}} = \{\mathcal{G}(\neg p(s) \vee \neg\alpha) \mid s \in S, \alpha \in AP_c, L_{\mathcal{A}}(s,\alpha) = \bot\}$.<br>If the KS is in state $s$ s.t. $L_{\mathcal{A}}(s,\alpha) = \bot$, the atomic proposition $\alpha$ is false. |
| $C_{REG} = \{\mathcal{G}(\neg p(s) \vee \neg p(s')) \mid s, s' \in S \text{ and } s \neq s'\}$<br>The KS is in at most one state at any time. |

where $C_{\mathcal{A}}$ are sets of LTL clauses obtained from the KS $\mathcal{A}$.[4] The set of clauses that encodes the KS is $C_{\mathcal{A}} = C_{KS} \cup C_{REG}$, where $C_{KS} = \{c_i\} \cup CR \cup CL_{\top,\mathcal{A}} \cup CL_{\bot,\mathcal{A}}$ and $c_i$, $CR$, $CL_{\top,\mathcal{A}}$ and $CL_{\bot,\mathcal{A}}$ are defined as specified in Table 2. Note that the clauses in $C_{\mathcal{A}}$ are defined on the set of atomic propositions $AP_S = AP_c \cup \{p(s) | s \in S\}$, i.e., $AP_S$ includes an additional atomic proposition $p(s)$ for each state $s$, which is true when the KS is in state $s$. The size of the encoding depends on the cardinality of $C_{\mathcal{A}}$ i.e., in the worst case, $1 + |S| + |S| \times |AP_c| + |S| \times |S|$.

GetUC. *Computation of the Unsatisfiable Core (UC) $\eta(C'_{\mathcal{A}} \cup \{\psi\})$ of $\eta(C_{\mathcal{A}} \cup \{\psi\})$*. Since the property $\psi$ is satisfied on $\mathcal{A}$, $\eta(C_{\mathcal{A}} \cup \{\psi\})$ is unsatisfiable and the computation of its UC core is performed by using the PLTLMUP approach [58]. Let $C = \{\varphi_1, \varphi_2, \ldots, \varphi_n\}$ be a set of LTL formulae, such that $\eta(C) = \bigwedge_{\varphi \in C} \varphi$ is unsatisfiable, then the function $\eta(C') = \text{GetUC}(\eta(C))$ returns an unsatisfiable core $\eta(C') = \bigwedge_{\varphi \in C'} \varphi$ of $\bigwedge_{\varphi \in C} \varphi$. In our case, since the property holds on the KS $\mathcal{A}$, $\text{GetUC}(\eta(C_{\mathcal{A}} \cup \{\psi\}))$ returns a subset of clauses $\eta(C'_{\mathcal{A}} \cup \{\psi\})$, where $C'_{\mathcal{A}} = C'_{KS} \cup C'_{REG}$ such that $C'_{KS} \subseteq C_{KS}$ and $C'_{REG} \subseteq C_{REG}$.

**Lemma 1.** *Let $\mathcal{A}$ be a KS and let $\psi$ be an LTL property. Let also $\eta(C_{\mathcal{A}} \cup \{\psi\})$ be the LTL formula computed in the step* Sys2LTL *of the algorithm. Then, any unsatisfiable core $\eta(C'_{\mathcal{A}} \cup \{\psi\})$ of $\eta(C_{\mathcal{A}} \cup \{\psi\})$ is such that $C'_{\mathcal{A}} \subseteq C_{\mathcal{A}}$.*

*Proof Sketch.* As the property $\phi$ is satisfied by $M$, the LTL formula $\eta(C_{\mathcal{A}} \cup \{\psi\})$, where $\psi = \mathtt{F}(\phi)$ must be unsatisfiable as discussed in the Section 3. Indeed, $\mathtt{F}(\phi)$ simply perform some proposition renaming on the negation of the formula $\psi$. As $C_{\mathcal{A}}$ encodes a KS, $\bigwedge_{c \in C_{\mathcal{A}}} c$ is satisfied. As such, the unsatisfiability is caused by the contradiction of some of the clauses in $C_{\mathcal{A}}$ and the property $\psi$, and as a consequence $\psi$ must be a part of the UC.

GetTP. *Analysis of $C'_{\mathcal{A}}$ and extraction of the topological proof.* The set $C'_{\mathcal{A}}$, where $C'_{\mathcal{A}} = C'_{KS} \cup C'_{REG}$, contains clauses regarding the KS ($C'_{KS}$ and $C'_{REG}$)

[4] Note that this formula is equivalent to $\phi_M \wedge \neg\phi$ used in Section 3 as $\phi_M$ is generated by the clauses in $C_{\mathcal{A}}$ and $\neg\phi$ from $\psi$.

Table 3: Rules to extract the TP-clauses from the UC LTL formula.

| LTL clause | TP clause | Type | LTL clause | TP clause | Type |
|---|---|---|---|---|---|
| $c_i = \bigvee_{s \in S_0} p(s)$ | $\langle S_0 \rangle$ | TPI | $\mathcal{G}(\neg p(s) \vee \neg \alpha)$ | $\langle s, \alpha, comp(L(s, \alpha)) \rangle$ | TPP |
| $\mathcal{G}(\neg p(s) \vee \mathcal{X}(\bigvee_{(s,s') \in R} p(s')))$ | $\langle s, T \rangle$ where $T = \{s' \mid (s, s') \in R\}$ | TPT | $\mathcal{G}(\neg p(s) \vee \overline{\alpha})$ | $\langle s, \alpha, comp(L(s, \alpha)) \rangle$ | TPP |
| $\mathcal{G}(\neg p(s) \vee \alpha)$ | $\langle s, \alpha, L(s, \alpha) \rangle$ | TPP | $\mathcal{G}(\neg p(s) \vee \neg \overline{\alpha})$ | $\langle s, \alpha, L(s, \alpha) \rangle$ | TPP |

and the property of interest ($\psi$) that made the formula $\eta(C'_{\mathcal{A}} \cup \{\psi\})$ unsatisfiable. Since we are interested in clauses related to the KS that caused unsatisfiability, we extract the topological proof $\Omega$, whose topological proof clauses are obtained from the clauses in $C'_{KS}$ as specified in Table 3. Since the set of atomic propositions of $\mathcal{A}$ is $AP_c = AP \cup \overline{AP}$, in the table we use $\alpha$ for propositions in $AP$ and $\overline{\alpha}$ for propositions in $\overline{AP}$.

The elements in $C'_{REG}$ are not considered in the TP computation as, given an LTL clause $\mathcal{G}(\neg p(s) \vee \neg p(s'))$, either state $s$ or $s'$ is constrained by other TP-clauses that will be preserved in the model revisions.

**Lemma 2.** *Let $\mathcal{A}$ be a KS and let $\psi$ be an LTL property. Let also $\eta(C_{\mathcal{A}} \cup \{\psi\})$ be the LTL formula computed in the step* Sys2LTL *of the algorithm, where $C_{\mathcal{A}} = C_{REG} \cup C_{KS}$, and let $\eta(C'_{\mathcal{A}} \cup \{\psi\})$ be an unsatisfiable core, where $C'_{\mathcal{A}} = C'_{REG} \cup C'_{KS}$. Then, if $\mathcal{G}(\neg p(s) \vee \neg p(s')) \in C'_{REG}$, either:*

*(i) there exists an LTL clause in $C'_{KS}$ that constrains state $s$ (or state $s'$); or*
*(ii) $\eta(C''_{\mathcal{A}} \cup \{\psi\})$, s.t. $C''_{\mathcal{A}} = C'_{\mathcal{A}} \setminus \{\mathcal{G}(\neg p(s) \vee \neg p(s'))\}$, is an UC of $\eta(C'_{\mathcal{A}} \cup \{\psi\})$.*

*Proof Sketch.* We indicate $\mathcal{G}(\neg p(s) \vee \neg p(s'))$ as $\tau(s, s')$. Assume per absurdum that conditions (i) and (ii) are violated, i.e., no LTL clause in $C'_{KS}$ constrains state $s$ or $s'$ and $\eta(C''_{\mathcal{A}} \cup \{\psi\})$ is not an unsatisfiable core of $\eta(C'_{\mathcal{A}} \cup \{\psi\})$. Since $\eta(C''_{\mathcal{A}} \cup \{\psi\})$ is not an unsatisfiable core of $\eta(C'_{\mathcal{A}} \cup \{\psi\})$, $\eta(C''_{\mathcal{A}} \cup \{\psi\})$ is satisfiable, as $C''_{\mathcal{A}} \subset C'_{\mathcal{A}}$. Since $\eta(C''_{\mathcal{A}} \cup \{\psi\})$ is satisfiable, $\eta(C'_{\mathcal{A}} \cup \{\psi\})$ s.t. $C'_{\mathcal{A}} = C''_{\mathcal{A}} \cup \{\tau(s, s')\}$ must also be satisfiable. Indeed, it does not exist any LTL clause that constrains state $s$ (or state $s'$) and, in order to generate a contradiction, the added LTL clause must generate it using the LTL clauses obtained from the LTL property $\psi$. This is a contradiction. Thus, conditions (i) and (ii) must be satisfied. □

The Analyze procedure in Algorithm 1 obtains a TP ([4], [6]) for a PKS by first computing the related optimistic or pessimistic approximation (i.e., a KS) and then exploiting the computation of the TP for such KS.

**Theorem 3.** *Let $M = \langle S, R, S_0, AP, L \rangle$ be a PKS, let $\phi$ be an LTL property, and let $x \in \{\top, ?\}$ be an element such that $[M \models \phi] = x$. If the procedure* Analyze*, applied to the PKS $M$ and the LTL property $\phi$, returns a TP $\Omega$, this is an $x$-TP for $\phi$ in $M$.*

*Proof Sketch.* Assume that the Analyze procedure returns the value $\top$ and a $\top$-TP. We show that every $\Omega$-related PKS $M'$ is such that $[M' \models \phi] \geq x$

(Definition 5). If ANALYZE returns the value $\top$, it must be that $M_{pes} \models^* \phi$ by Lines 5 and 7 of Algorithm 1. Furthermore, by Line 7, $\psi = \mathtt{F}(\phi)$ and $\mathcal{A} = M_{pes}$.

Let $N = \langle S_N, R_N, S_{0,N}, AP_N, L_N \rangle$ be a PKS $\Omega$-related to $M$. Let $\eta(C_{\mathcal{A}} \cup \{\psi\})$ be the LTL formula associated with $\mathcal{A}$ and $\psi$ and let $\eta(C_{\mathcal{B}} \cup \{\psi\})$ be the LTL formula associated with $\mathcal{B} = N_{pes}$ and $\psi$. Let us consider an UC $\eta(C'_{\mathcal{A}} \cup \{\psi\})$ of $\eta(C_{\mathcal{A}} \cup \{\psi\})$, where $C'_{\mathcal{A}} = C'_{KS} \cup C'_{REG}$, $C'_{KS} \subseteq C_{KS}$ and $C'_{REG} \subseteq C_{REG}$. We show that $C'_{\mathcal{A}} \subseteq C_{\mathcal{B}}$, i.e., the UC is also an UC for the LTL formula associated with the approximation $\mathcal{B}$ of the PKS $N$.

- $C'_{\mathcal{A}} \subseteq C_{\mathcal{B}}$, i.e., $(C'_{KS} \cup C'_{REG}) \subseteq C_{\mathcal{B}}$. By Lemma 2 we can avoid considering $C'_{REG}$. By construction (see Line 2 of Algorithm 2) any clause $c \in C'_{KS}$ belongs to one rule among $CR$, $CL_{pes,\top}$, $CL_{pes,\bot}$ or $c = c_i$:
  - if $c = c_i$ then, by the rules in Table 3, there is a TPI-clause $\{S_0\} \in \Omega$. By Definition 4, $S_0 = S'_0$. Thus, $c_i \in C_{\mathcal{B}}$ since $N$ is $\Omega$-related to $M$.
  - if $c \in CR$ then, by rules in Table 3, there is a TPT-clause $\langle s, T \rangle \in \Omega$ where $s \in S$ and $T \subseteq R$. By Definition 4, $T = \{s' \in S' | (s, s') \in R'\}$. Thus, $c \in C_{\mathcal{B}}$ since $N$ is $\Omega$-related to $M$.
  - if $c \in CL_{\mathcal{A},\top}$ or $c \in CL_{\mathcal{A},\bot}$, by rules in Table 3, there is a TPP-clause $\langle s, \alpha, L(s, \alpha) \rangle \in \Omega$ where $s \in S$ and $\alpha \in AP$. By Definition 4, $L'(s, \alpha) = L(s, \alpha)$. Thus, $c \in C_{\mathcal{B}}$ since $N$ is $\Omega$-related to $M$.

Since $N$ is $\Omega$-related to $M$, it has preserved the elements of $\Omega$. Thus $\eta(C'_{\mathcal{A}} \cup \{\psi\})$ is also an UC of $C_{\mathcal{B}}$. It follows that $[N \models \phi] = \top$.

The proof from the case in which ANALYZE procedure returns the value ? and a ?-TP can be derived from the first case. □

**RE-CHECK (4).** Let $M = \langle S, R, S_0, AP, L \rangle$ be a PKS. The RE-CHECK algorithm verifies whether a revision $M'$ of $M$ is an $\Omega$-revision. Let $\Omega$ be an $x$-TP (10) for $\phi$ in $M$, and let $M' = \langle S', R', S'_0, AP', L' \rangle$ be a revision of $M$ (8). The RE-CHECK algorithm returns `true` if and only if the following holds:

- $AP \subseteq AP'$;
- for every TPP-clause $\langle s, \alpha, v \rangle \in \Omega$, $s \in S'$, $v = L'(s, \alpha)$;
- for every TPT-clause $\langle s, T \rangle \in \Omega$, $s \in S'$, $T \subseteq S'$, $T = \{s' \in S' | (s, s') \in R'\}$;
- for every TPI-clause $\langle S_0 \rangle \in \Omega$, $S_0 = S'_0$.

These conditions can be verified by a simple syntactic check on the PKS.

**Lemma 3.** *Let $M = \langle S, R, S_0, AP, L \rangle$ and $M' = \langle S', R', S'_0, AP', L' \rangle$ be two PKSs and let $\Omega$ be an $x$-TP. The* RE-CHECK *algorithm returns* `true` *if and only if $M'$ is $\Omega$-related to $M$.*

*Proof Sketch.* Since $M'$ is $\Omega$-related to $M$, the conditions of Definition 4 hold. Each of these conditions is a condition of the RE-CHECK algorithm. Thus, if $M'$ is $\Omega$-related to $M$, the RE-CHECK returns `true`. Conversely, if RE-CHECK returns `true`, each condition of the algorithm is satisfied and, since each of these conditions corresponds to a condition of Definition 4, $M'$ is $\Omega$-related to $M$. □

This Lemma allows us to prove the following Theorem.

Table 4: Properties considered in the evaluation

| | |
|---|---|
| $\phi_1$: | $\mathcal{G}(\neg OFFHOOK) \vee (\neg OFFHOOK\ \mathcal{U}\ CONNECTED)$ |
| $\phi_2$: | $\neg OFFHOOK\ \mathcal{W}\ (\neg OFFHOOK \wedge CONNECTED)$ |
| $\phi_3$: | $\mathcal{G}(CONNECTED \rightarrow ACTIVE)$ |
| $\phi_4$: | $\mathcal{G}(OFFHOOK \wedge ACTIVE \wedge \neg CONNECTED \rightarrow \mathcal{X}(ACTIVE))$ |
| $\phi_5$ | $\mathcal{G}(CONNECTED \rightarrow \mathcal{X}(ACTIVE))$ |
| $\psi_1$: | $\mathcal{G}(CONNECTED \rightarrow ACTIVE)$ |
| $\psi_2$: | $\mathcal{G}(CONNECTED \rightarrow \mathcal{X}(ACTIVE))$ |
| $\psi_3$: | $\mathcal{G}(CONNECTED) \vee (CONNECTED\ \mathcal{U}\ \neg OFFHOOK)$ |
| $\psi_4$: | $\neg CONNECTED\ \mathcal{W}\ (\neg CONNECTED \wedge OFFHOOK)$ |
| $\psi_5$: | $\mathcal{G}(CALLEE\_SEL \rightarrow OFFHOOK)$ |
| $\eta_1$: | $\mathcal{G}((OFFHOOK \wedge CONNECTED) \rightarrow \mathcal{X}(OFFHOOK \vee \neg CONNECTED))$ |
| $\eta_2$: | $\mathcal{G}(CONNECTED) \vee (CONNECTED\ \mathcal{W}\ \neg OFFHOOK)$ |
| $\eta_3$: | $\neg CONNECTED\ \mathcal{W}\ (\neg CONNECTED \wedge OFFHOOK)$ |
| $\eta_4$: | $\mathcal{G}(CALLEE\_FREE \vee LINE\_SEL)$ |
| $\eta_5$: | $\mathcal{G}(\mathcal{X}(OFFHOOK) \wedge \neg CONNECTED)$ |

**Theorem 4.** *Let $M$ be a PKS, let $\phi$ be a property, let $\Omega$ be an $x$-TP for $\phi$ in $M$ where $x \in \{\top, ?\}$, and let $M'$ be a revision of $M$. The* RE-CHECK *algorithm returns* `true` *if and only if $M'$ is an $\Omega$-revision of $M$.*

*Proof Sketch.* By applying Lemma 3, the RE-CHECK algorithm returns `true` if and only if $M'$ is $\Omega$-related to $M$. By Definition 6, since $\Omega$ is an $x$-TP, the RE-CHECK algorithm returns `true` if and only if $M'$ is an $\Omega$-revision of $M$. □

The ANALYSIS and RE-CHECK algorithms assume that the three-valued LTL semantics is considered. While the thorough LTL semantics [10] has been shown to provide an evaluation of formulae that better reflects the natural intuition, the two semantics coincide in the case of self-minimizing LTL formulae. In this case, our results are correct also w.r.t. the thorough semantics. Note that, as shown in [24], most practically useful LTL formulae are self-minimizing. Future work will consider how to extend the ANALYSIS and RE-CHECK to completely support the thorough LTL semantics.

## 6 Evaluation

We implemented TOrPEDO as a Scala stand alone application and made it available online [62]. We evaluated how the ANALYSIS helps in creating models revisions and how frequently running the RE-CHECK algorithm allows the user to avoid the re-execution of the ANALYSIS algorithm from scratch.

We considered a set of example PKSs proposed in the literature to evaluate the $\chi$*Chek* [20] model checker and defined a set of properties (see Table 4) inspired by the original properties and based on the LTL property patterns [18].[5]

**ANALYSIS support (2).** We checked how the size of the proofs compares w.r.t. the size of the original models. Intuitively, since the proofs represent constraints

[5] The original properties used in the examples were specified in Computation Tree Logic (CTL), which is currently not supported by TOrPEDO.

Table 5: Cardinalities $|S|$, $|R|$, $|AP|$, $|?|$, and $|M|$ are those of the evaluated model $M$. $|\Omega_p|_x$ is the size of proof $\Omega_p$ for a property $p$; $x$ indicates if $\Omega_p$ is a $\top$-TP or a ?-TP.

| | ANALYSIS | | | | | | | | | | RE-CHECK | | | | |
|---|---|---|---|---|---|---|---|---|---|---|---|---|---|---|---|
| Model | $\vert S\vert$ | $\vert R\vert$ | $\vert AP\vert$ | $\vert ?\vert$ | $\vert M\vert$ | $\vert\Omega_{\phi_1}\vert$ | $\vert\Omega_{\phi_2}\vert$ | $\vert\Omega_{\phi_3}\vert$ | $\vert\Omega_{\phi_4}\vert$ | $\vert\Omega_{\phi_5}\vert$ | $\phi_1$ | $\phi_2$ | $\phi_3$ | $\phi_4$ | $\phi_5$ |
| callee-1 | 5 | 15 | 3 | 7 | 31 | $7_?$ | $9_?$ | $21_?$ | $23_?$ | $23_?$ | - | - | - | - | - |
| callee-2 | 5 | 15 | 3 | 4 | 31 | $7_?$ | $9_?$ | $21_?$ | $22_\top$ | × | ✓ | ✓ | ✓ | ✓ | ✗ |
| callee-3 | 5 | 15 | 3 | 2 | 31 | $7_?$ | $9_?$ | $21_?$ | $23_\top$ | × | ✓ | ✓ | ✓ | ✓ | - |
| callee-4 | 5 | 15 | 3 | 0 | 31 | × | × | $23_\top$ | $21_\top$ | × | ✗ | ✗ | ✓ | ✓ | - |
| Model | $\vert S\vert$ | $\vert R\vert$ | $\vert AP\vert$ | $\vert ?\vert$ | $\vert M\vert$ | $\vert\Omega_{\psi_1}\vert$ | $\vert\Omega_{\psi_2}\vert$ | $\vert\Omega_{\psi_3}\vert$ | $\vert\Omega_{\psi_4}\vert$ | $\vert\Omega_{\psi_5}\vert$ | $\psi_1$ | $\psi_2$ | $\psi_3$ | $\psi_4$ | $\psi_5$ |
| caller-1 | 6 | 21 | 5 | 4 | 52 | $28_?$ | × | $2_\top$ | $9_?$ | $28_?$ | - | - | - | - | - |
| caller-2 | 7 | 22 | 5 | 4 | 58 | $30_?$ | × | $2_\top$ | $9_?$ | $30_?$ | ✓ | - | ✓ | ✓ | ✓ |
| caller-3 | 6 | 19 | 5 | 1 | 50 | $26_\top$ | $28_\top$ | $2_\top$ | $11_\top$ | $26_\top$ | ✓ | - | ✓ | ✓ | ✓ |
| caller-4 | 6 | 21 | 5 | 0 | 52 | $28_\top$ | × | $2_\top$ | $9_\top$ | $28_\top$ | ✓ | ✗ | ✓ | ✓ | ✓ |
| Model | $\vert S\vert$ | $\vert R\vert$ | $\vert AP\vert$ | $\vert ?\vert$ | $\vert M\vert$ | $\vert\Omega_{\eta_1}\vert$ | $\vert\Omega_{\eta_2}\vert$ | $\vert\Omega_{\eta_3}\vert$ | $\vert\Omega_{\eta_4}\vert$ | $\vert\Omega_{\eta_5}\vert$ | $\eta_1$ | $\eta_2$ | $\eta_3$ | $\eta_4$ | $\eta_5$ |
| caller-callee-1 | 6 | 30 | 6 | 30 | 61 | $37_?$ | $2_\top$ | $15_?$ | $37_?$ | × | - | - | - | - | - |
| caller-callee-2 | 7 | 35 | 6 | 36 | 78 | $43_?$ | $2_\top$ | $18_?$ | $43_?$ | × | ✓ | ✓ | ✓ | ✓ | - |
| caller-callee-3 | 7 | 45 | 6 | 38 | 88 | $53_?$ | $2_\top$ | $53_?$ | $53_?$ | $53_?$ | ✓ | ✓ | ✓ | ✓ | - |
| caller-callee-4 | 6 | 12 | 4 | 0 | 42 | × | × | × | $19_\top$ | × | ✗ | ✗ | ✗ | ✓ | ✗ |

that, if satisfied, ensure that the property is not violated (or possible violated), the smaller are the proofs the more flexibility the designer has, as more elements can be changed during the revision. The size of a PKS $M = \langle S, R, S_0, AP, L\rangle$ was defined as $|M| = |AP| * |S| + |R| + |S_0|$. The size of a proof $\Omega$ was defined as $|\Omega| = \sum_{c\in\Omega} |c|$ where: $|c| = 1$ if $c = \langle s, \alpha, v\rangle$; $|c| = |T|$ if $c = \langle s, T\rangle$, and $|c| = |S_0|$ if $c = \langle S_0\rangle$. Table 5 summarizes the obtained results (columns under the label ANALYSIS). We show the cardinalities $|S|$, $|R|$ and $|AP|$ of the sets of states, transitions, and atomic propositions of each considered PKS $M$, the number $|?|$ of couples of a state $s$ with an atomic proposition $\alpha$ such that $L(s, \alpha) =?$, the total size $|M|$ of the model, and the size $|\Omega_p|_x$ of the proofs, where $p$ indicates the considered LTL property and $x$ indicates whether $p$ is satisfied ($x = \top$) or possibly satisfied ($x =?$). Proofs are $\approx 60\%$ smaller than their respective initial models. Thus, we conclude that the proofs are significantly coincise w.r.t. the original model enabling a flexible design.

**RE-CHECK support (3).** We checked how the results output by the RE-CHECK algorithm were useful in producing PKSs revisions. To evaluate the usefulness we assumed that, for each category of examples, the designer produced revisions following the order specified in Table 5. The columns under the label RE-CHECK contain the different properties that have been analyzed for each category. A cell contains ✓ if the RE-CHECK was passed by the considered revised model, i.e., a `true` value was returned by the RE-CHECK algorithm, ✗ otherwise. The *dash* symbol - is used when the model of the corresponding line is not a revision (i.e., the first model of each category) or when the observed property was false in the previous model, i.e., an $x$-TP was not produced. We inspected the results produced by the RE-CHECK algorithm to evaluate their benefit in verifying if revisions were violating the proofs. Table 5 shows that, in $\approx 32\%$ of the cases, the TOrPEDO re-check notified the designer that the proposed revision violated some of the clauses contained in the $\Omega$-proof, while in $\approx 78\%$ the re-check allowed designers to avoid re-runnning the analysis (and thus the model checker).

**Scalability.** The analysis phase of TOrPEDO combines three-valued model checking and UCs computation, therefore its scalability improves as the performance of frameworks enhances. Three-valued model checking is as expensive as classical model checking [9], i.e., it is linear in the size of the model and exponential in the size of the property. UCs computation is FPSPACE complete [55]. In our cases running TOrPEDO required on average 8.1s and for the callee examples, 8.2s for the caller examples, and 7.15s for the caller-callee examples.[6] However, while model checking is currently supported by very efficient techniques, UCs computation of LTL formulae is still far from being applicable in complex scenarios. For example, we manually designed an additional PKS with 10 states and 5 atomic propositions and 26 transitions and defined a property satisfied by the PKS and with a $\top$-TP proof that requires every state of the PKS to be constrained by a TPP-clause. We run TOrPEDO and measure the time required to compute this proof. Computing the proof required 1m33s. This results show that TOrPEDO has a limited scalability due to the low efficiency of the procedure that extracts the unsatisfiable core. For an analysis of the scalability of the extraction of the unsatisfiable core the interested reader can refer to [58]. We believe that reporting the current lack of FM techniques to support the proposed framework (that, as just discussed, is effective in our preliminary evaluation), is a further contribution of this paper.

## 7 Related work

Partial knowledge has been considered in requirement analysis and elicitation [46,45,38,13], in novel robotic planners [40,41,43], software models [66,65,22,1], and testing [15,63,67]. Several researchers analyzed the model checking problem for partially specified systems [44,12], considering both three-valued [37,25,9,10,28] and multi-valued [30,11] semantics. Other works apply model checking to incremental program development [33,6]. However, all these model checking approaches do not provide an *explanation* on why a property is satisfied, by means of a *certificate* or *proof*. Although several works have tackled this problem [4,60,50,49,29,16], differently from this work, they mostly aim to automate proof reproducibility.

Tao and Li [61] propose a theoretical solution to model repair: the problem of finding the minimum set of states in a KS which makes a formula satisfiable. However, the problem is different from the one addressed in this paper. Furthermore, the framework is only theoretical and based on complete systems.

Approaches were proposed in the literature to provide explanations by using different artifacts. For example, some works proposed using witnesses. A witness

[6] Processor: 2,7 GHz Quad-Core Intel Core i7, Memory: 16 GB 2133 MHz LPDDR3.

is a path of the model that satisfies a formula of interest [7,34,48]. Other works (e.g., [31,59]) studied how to enrich counterexamples with additional information in a way that allows better understanding the property violation. Work has also been done to generate abstractions of the counterexamples that are easier to understand (e.g., [21]). Alur et al. [2] analyzed the problem of synthesizing a controller that satisfies a given specification. When the specification is not realizable, a counter-strategy is returned as a witness. Pencolé et al. [51] analyzed model consistency, i.e., the problem of checking whether the system run-time behaviour is consistent with a formal specification. Bernasconi et al. [4] proposed an approach that combines model checking and deductive proofs in a multi-valued context. The notion of topological proof proposed in this work is substantially different from the notion of deductive proof.

Some works (e.g., [52,54]) considered how to understand why a property is unsatisfiable. This problem is different from the one considered in this paper.

Approaches that detect unsatisfiable cores of propositional formulae were proposed in the literature [47,39,17,32,57]. Understanding whether these approaches can be re-used to develop more efficient techniques to detect the unsatisfiable cores of LTL formulae is definitely an interesting future work direction, which deserves to be considered in a separate work since it is far from trivial.

## 8 Conclusions

We have proposed TOrPEDO, an integrated framework that supports the iterative creation of model revisions. The framework provides a guide for the designer who wishes to preserve slices of her model that contribute to satisfy fundamental requirements while other parts of the model are modified. For these purposes, the notion of topological proof has been formally and algorithmically described. This corresponds to a set of constraints that, if kept when changing the proposed model, ensure that the behavior of the model w.r.t. the property of interest is preserved. Our Lemmas and Theorems prove the soundness of our framework, i.e., how it preserves correctness in the case of PKS and LTL. The proposed framework can be used as baseline for other FM frameworks, and can be extended by considering other modeling formalisms that can be mapped onto PKSs.

TOrPEDO was evaluated by showing the effectiveness of the ANALYSIS and RE-CHECK algorithms included in the framework. Results showed that proofs are smaller than the original models, and can be verified in most of the cases using a simple syntactic check, paving the way for an extensive evaluation on real case scenarios. However, the scalability of existing tools, upon which TOrPEDO is based, is not sufficient to efficiently support the proposed framework when bigger models are considered.

**Acknowledgments.** This work has received funding from the European Research Council under the European Union's Horizon 2020 research and innovation programme (grant agreement No 694277).
